# Supplementary material for: Socioeconomic Status and Interest in Genetic Testing in a US-Based Sample
Source: Healthcare (Basel). 2022 May 10;10(5):880. doi: 10.3390/healthcare10050880 (PMC9141316; doi:10.3390/healthcare10050880)
Supplement: Supplementary file 1 [file healthcare-10-00880-s001.zip › healthcare-1692426-supplementary.pdf]

Supplemental S1: MacArthur Scale of Subjective Social Status

79. Think of this ladder as representing where people stand in the United States.

At the **top** of the ladder (10) are the people who are the best off – those who have the most money, the most education and the most respected jobs. At the **bottom** (1) are the people who are the worst off – who have the least money, least education, and the least respected jobs or no job. The higher up you are on this ladder, the closer you are to the people at the very top; the lower you are, the closer you are to the people at the very bottom.

Where would you place yourself on this ladder?

Select a number 1 – 10 (1 representing people who are the worst off and 10 people who are the best off) on the ladder where you think you stand at this time in your life, relative to other people in the United States.

- ○ 10
- ○ 9
- ○ 8
- ○ 7
- ○ 6
- ○ 5
- ○ 4
- ○ 3
- ○ 2
- ○ 1

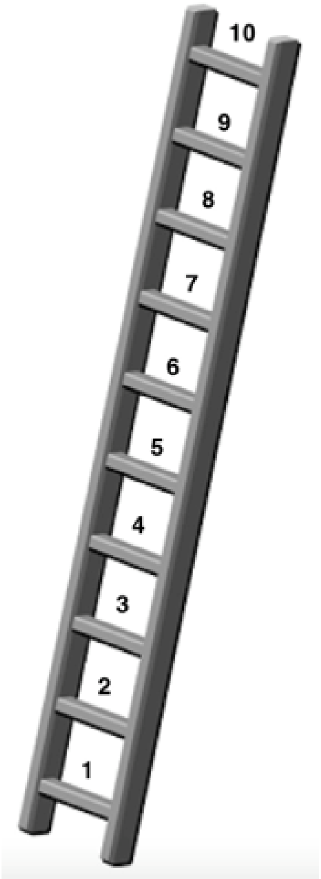

Supplemental S2: Measure of Interest in Pursuing Genetic Testing from Patient Baseline Survey

Everyone has some risk of developing cancer. Cancer is usually caused by gene mutations that happen randomly, but sometimes the mutation is hereditary (passed from a parent to their child). People who carry a hereditary mutation do not always get cancer, but their risk is higher than average.

1. How many first-degree relatives do you have? (First-degree relatives are those directly related to you – your parents, children, and siblings.)

____

2. How many of your first-degree relatives have had genetic testing for cancer?

____

3. Have you ever had genetic testing for cancer?

○ Yes

○ No

○ Uncertain

4. How many of your first-degree relatives have had cancer?

____

5. Have you ever had cancer?

○ Yes

○ No

○ Uncertain

6. How many of your first-degree relatives have died from cancer?

____

7. If your personal and familial history suggested you were at high risk for cancer, how interested would you be in having genetic testing?

Not at all interested	A little bit interested	Moderately interested	Very interested	Extremely interested
○	○	○	○	○

Below is a list of reasons someone might give for having genetic testing for hereditary cancer. For each item, please indicate if you see it as a reason to have genetic testing, with 1=strongly disagree and 5=strongly agree.

I would get genetic testing to…

Strongly Disagree Disagree Uncertain Agree Strongly Agree

8. Provide reassurance that I will not get cancer. ○ ○ ○ ○ ○

9. Establish my personal risk of developing cancer. ○ ○ ○ ○ ○

10. Determine whether more frequent / different cancer prevention measures could reduce my personal risk of developing cancer. ○ ○ ○ ○ ○

11. Help my family members understand their risk of developing cancer. ○ ○ ○ ○ ○

12. Determine whether my family members should have genetic testing. ○ ○ ○ ○ ○

Please indicate whether each of the following statements would make you more likely to have genetic testing for hereditary cancer, with 1=strongly disagree and 5=strongly agree.

I would be interested in genetic testing if…

Strongly Disagree Disagree Uncertain Agree Strongly Agree

13. My doctor recommended it. ○ ○ ○ ○ ○

14. The information might affect my health. ○ ○ ○ ○ ○

15. The information might affect my family member's health. ○ ○ ○ ○ ○

16. The test was free/low cost. ○ ○ ○ ○ ○

17. Please share any thoughts you have about reasons for or against having genetic testing.

____
